# Distortions of political bias in crowdsourced misinformation flagging

**DOI:** 10.1098/rsif.2020.0020

**Published:** 2020-06-10

**Authors:** Michele Coscia, Luca Rossi

**Affiliations:** IT University of Copenhagen, Kobenhavn, Denmark

**Keywords:** social media, social networks, content policing, flagging, fake news, echo chambers

## Abstract

Many people view news on social media, yet the production of news items online has come under fire because of the common spreading of misinformation. Social media platforms police their content in various ways. Primarily they rely on crowdsourced ‘flags’: users signal to the platform that a specific news item might be misleading and, if they raise enough of them, the item will be fact-checked. However, real-world data show that the most flagged news sources are also the most popular and—supposedly—reliable ones. In this paper, we show that this phenomenon can be explained by the unreasonable assumptions that current content policing strategies make about how the online social media environment is shaped. The most realistic assumption is that confirmation bias will prevent a user from flagging a news item if they share the same political bias as the news source producing it. We show, via agent-based simulations, that a model reproducing our current understanding of the social media environment will necessarily result in the most neutral and accurate sources receiving most flags.

## 1. Introduction

Social media have a central role to play in the dissemination of news [1]. There is a general concern about the low quality and reliability of information viewed online: researchers have dedicated increasing amounts of attention to the problem of so-called fake news [2–4]. Given the current ecosystem of news consumption and production, misinformation should be understood within the complex set of social and technical phenomena underlying online news propagation, such as echo chambers [5–10], platform-induced polarization [11,12] and selective exposure [13,14].

Over the years two main approaches have emerged to try to address the problem of fake news by limiting its circulation: a technical approach and an expert-based approach. The technical approach aims at building predictive models able to detect misinformation [15,16]. This is often done using one or more features associated with the message, such as content (through natural language processing (NLP) approaches [17]), source reliability [18] or network structure [19]. While these approaches have often produced promising results, the limited availability of training data as well as the unavoidable subjectivity involved in labelling a news item as fake [20,21] constitute a major obstacle to wider development.

The alternative expert-based approach consists of a fact-checker on the specific topic that investigates and evaluates each claim. While this could be the most accurate way to deal with misinformation, given the amount of news that circulates on social media every second, it is hard to imagine how this could scale to the point of being effective. For this reason, the dominant approach, which has recently also been adopted by Facebook,^1^ is based on a combination of methods that first use computationally detected crowd signals, often constituted by users *flagging* what they consider fake or misleading information, and then assigning selected news items to external professional fact-checkers for further investigation [22,23]. Although flagging-based systems remain, to the best of our knowledge, widely used, many authors have questioned their reliability, showing how users can flag news items for reasons other than the ones intended [24,25]. Recently, researchers proposed methods to identify reliable users and improve, in that way, the quality of the crowd signal [20,23].

Regardless of the ongoing efforts, fake news and misleading information still pollute online communications and no immediate solution seems to be available. In 2018, Facebook released, through the Social Science One initiative, the Facebook URL Shares dataset [26], a preview of the larger dataset released recently.^2^ The dataset contains the web page addresses (URLs) shared by at least 20 unique accounts on Facebook between January 2017 and June 2018. Together with the URLs, the dataset also details whether the specific link had been sent to the third-party fact-checkers that collaborate with Facebook.

We accessed the most shared links in the Italian subset, which revealed some curious patterns and inspired the present work. We exclusively use this dataset for the motivation and validation of our analysis, leaving the use of the newer full dataset for future work.

**Figure 1.**
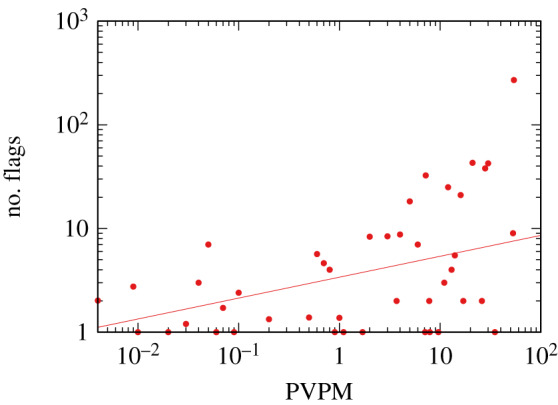
The relationship between the web traffic of a website (*x*-axis) and the number of flags it received on Facebook (*y*-axis). Traffic is expressed in PPVM, which indicates what fraction of all the page views by Alexa toolbar users go to a particular site.

Table 1 shows the top 10 most reported domains, which are exclusively major national newspapers, news sites and a satirical website. A further analysis of the data reveals, as figure 1 shows, a positive correlation ($y=\beta x^{\alpha }$ fit, with slope *α* = 0.2, scale *β* = 1.22 and *p* < 0.001^3^) between a source’s popularity and the number of times a domain has been checked by Facebook’s third-party fact-checkers. We measure the popularity of the source through Alexa’s (https://www.alexa.com) *page views per million users* (PVPM). It is worth observing that all the news reported in the top 10 most reported domains have been fact-checked as true legitimate news (with the obvious exception of the satirical website, which was fact-checked as satire).

**Table 1.** The top 10 most flagged domains among the Italian links shared on the Facebook URL Shares dataset.

	domain	reported	PVPM	type
1	repubblica.it	270.00	54.00	national newspaper
2	ilfattoquotidiano.it	85.00	21.00	national newspaper
3	corriere.it	83.00	30.00	national newspaper
4	fanpage.it	49.00	5.00	national news site
5	ansa.it	47.00	12.00	national news site
6	huffingtonpost.it	40.00	7.20	national news site
7	ilmessaggero.it	34.00	2.00	national newspaper
8	ilsole24ore.com	32.00	4.00	national newspaper
9	lercio.it	29.00	3.00	satire
10	tgcom24.mediaset.it	28.00	28.00	national news site

These observations create the background for the present paper. Our hypothesis is that users are polarized and that polarization is an important driver of the decision of whether to flag or not a news item: a user will only flag it if it is not perceived truthful enough *and* if it has a significantly different bias from that of the user (polarity). Sharing the same bias would act against the user’s flagging action. Thus, we introduce a model of online news flagging that we call the ‘bipolar’ model, since we assume for simplicity that there are only two poles—roughly corresponding to ‘liberal’ and ‘conservative’ in the US political system. The bipolar model of news-flagging attempts to capture the main ingredients that we observe in empirical research on fake news and disinformation—echo chambers, confirmation bias, platform-induced polarization and selective exposure. We show how the proposed model provides a reasonable explanation of the patterns that we observe in Facebook data.

The current crowdsourced flagging systems seem to assume a simpler flag-generating model. Despite being somehow similar to the bipolar model we propose, in this simple case the model does not account for users’ polarization, thus we will call it the ‘monopolar’ model. In the monopolar model, users do not gravitate around two poles and perceived truthfulness constitutes the only parameter. Users flag news items only if they perceive an excessive ‘fakeness’ of the news item, depending of their degree of scepticism. We show how the monopolar model relies on unrealistic expectations and that it is unable to reproduce the observed flag-generating patterns.

Lastly, we test the robustness of the bipolar model against various configurations of the underlying network structure and the actors’ behaviour. We show, on the one hand, how the model is always able to explain the observed flagging phenomenon and, on the other hand, that a complex social network structure is a core element of the system.

## 2. Methods

In this section, we present the main model on which we base the results of this paper. It is possible to understand the bipolar and monopolar models as a single model with or without users’ polarization. However, a user’s polarization has a significant impact on the results, and it seriously affects the social network underlying the flagging and propagation processes. For these reasons, in the paper, we will refer to them as two different models with two different names, which makes the comparison easier to grasp.

In the following, we start by giving a general overview of the bipolar model (§2.1). In the subsequent sections, we provide the model details, motivating each choice on the basis of real-world data. We conclude by showing the crucial differences between the bipolar and monopolar models (§2.5).

We note that our model shares some commonalities with the bounded confidence model [27].

### 2.1. Model overview

**Figure 2.**
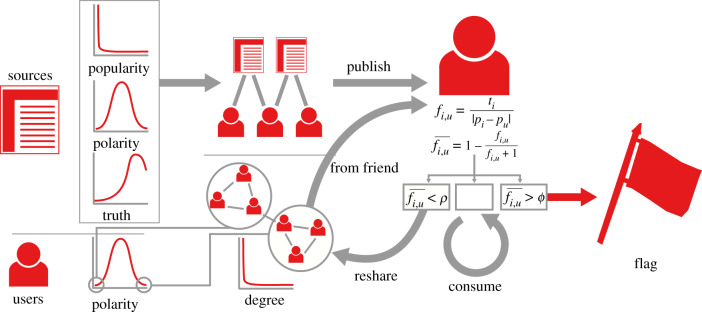
The overview of the bipolar model. From left to right, we show: the characteristics of the agents (source’s polarities, popularity and truthfulness; and user’s polarity); the model’s structures (the bipartite source–user follower network and the unipartite user–user social network); and the agents’ actions (source publishing and users resharing, consuming and flagging news items).

Figure 2 shows a general depiction of the bipolar model. In the bipolar model, we have two kinds of agents: news sources and users.

News sources are characterized by three values: popularity, polarity and truthfulness. The popularity distributes broadly: there are a few big players with a large following while the majority of sources are followed by only a few users. The polarity distributes quasi-normally. Most sources are neutral and there are progressively fewer and fewer sources that are more polarized. Truthfulness is linked to polarity, with more polarized sources tending to be less truthful. This implies that most news sources are truthful, and less trustworthy sources are more and more rare. Each news item has the same polarity and truthfulness values as the news source publishing it.

Users only have polarity. The polarity of the users distributes in the same way as that of the news sources. Most users are moderate and extremists are progressively more rare. Users follow news sources, preferentially those of similar polarity (selective exposure). Users embed in a social network, preferentially being friends of other users of similar polarity (homophily).

A user can see a news item if the item is either published by a source the user is following or reshared by one of their friends. In either case, the user can do one of three things:

1. reshare—if the polarity of the item is sufficiently close to their own *and* the item is sufficiently truthful;
2. flag—if the polarity of the item is sufficiently different from their own *or* the item is not truthful enough;
3. consume—in all other cases, meaning that the item does not propagate and nor is it flagged.

We expect the bipolar model to produce mostly flags in the moderate and truthful part of the spectrum. We base this expectation on the following reasoning. Since most news sources are moderate and truthful, the few very popular sources are overwhelmingly more likely to be moderate and truthful. Thus we will see more moderate and truthful news items, which are more likely to be reshared. This resharing activity will cause the news items published by the moderate and truthful news sources to be shared to the polarized parts of the network. Here, given that the difference between the polarization of the user and the polarization of the source plays a role in flagging even relatively truthful items, moderate and truthful news items are likely to be flagged.

Polarized and untruthful items, on the other hand, are unlikely to be reshared. Because of the polarization homophily that characterizes the network structure, they are unlikely to reach the more moderate parts of the network. If polarized items are not shared, they cannot be flagged. A neutral item is more likely to be shared, and thus could reach a polarized user, who would flag it. Thus, most flags will hit moderate and truthful news items, rendering the whole flagging mechanism unsuitable for discovering untruthful items.

### 2.2. Agents

In this section, we detail how we build the main agents in our model: the news sources and the users.

As mentioned previously, news sources have a certain popularity. The popularity of a news source is the number of users following it. We generate the source popularity distribution as a power law. This means that the vast majority of news sources have a single follower, while the most popular sources have thousands of followers.

**Figure 3.**
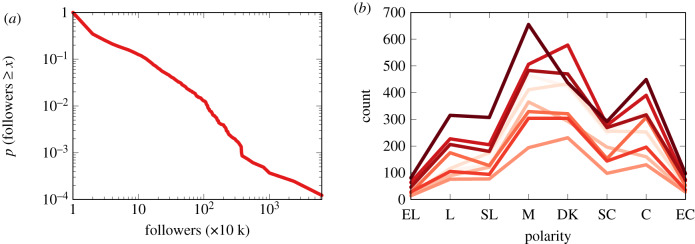
(*a*) The cumulative distribution of source popularity on Facebook in our dataset: the probability (*y*-axis) of a page to have a given number of followers or more (*x*-axis). (*b*) The polarity distribution in the USA from 1994 (light) to 2016 (dark). Biannual observation, except for missing years 2006, 2010 and 2014. EL, extremely liberal; L, liberal; SL, slightly liberal; M, moderate; DK, don’t know; SC, slightly conservative; C, conservative; EC, extremely conservative.

This is supported by real-world data. Figure 3*a* shows the complement cumulative distribution of the number of followers of Facebook pages. These data come from CrowdTangle.^4^ As we can see, the distribution has a long tail: two out of three Facebook pages have 10 000 followers or fewer. The most popular pages are followed by more than 60 million users.

As for the user and source polarities (*p*_*u*_ and *p*_*i*_), we assume that they distribute quasi-normally. We create a normal distribution with average equal to zero and standard deviation equal to 1. Then we divide it by its maximum absolute value to ensure that the distribution fully lies between −1 and 1. In this way we ensure that most users are moderates; more extreme users/sources are progressively more rare, at both ends of the spectrum.

This is also supported by the literature [28] and by real-world data. Figure 3*b* shows the distribution of political leaning in the USA across time [29], collected online.^5^ These data were collected by surveying a representative sample of the US electorate via phone and face-to-face interviews.

While not perfectly normally distributed, the data show that the majority of Americans either feel they are moderate or do not know to which side they lean. ‘Moderate’ or ‘don’t know’ is always the mode of the distribution, and their combination is always the plurality option.

Finally, sources have a degree of truthfulness *t*_*i*_. Here, we make the assumption that this is correlated with the news source’s polarity. The more a source is polarized, the less it is interested in the actual truth. A polarized source wants to bring readers onto their side, and their ideology clouds their best judgement of truthfulness. This reasonable assumption is also supported by the literature [30].

Mathematically, this means that *t*_*i*_ = 1 − |*p*_*i*_| + *ε*, with −0.05 ≤ *ε* ≤ 0.05 being extracted uniformly at random, ensuring then that *t*_*i*_ remains between 0 and 1 by capping it to these values.

### 2.3. Structures

There are two structures in the model: the user–source bipartite network and the user–user social network.

#### 2.3.1. User–source network

The user–source network connects users to the news sources they are following. This is the primary channel through which users are exposed to news items.

We fix the degree distribution of the sources to be a power law, as we detailed in the previous section. The degree distribution of the user depends on the other rules of the model. There is a certain number of users with degree zero in this network. These users do not follow any news source and only react to what is shared by their circle of friends. We think this is reasonably realistic.

We connect users to sources to maximize polarity homophily. The assumption is that users will follow news organizations sharing their polarity. This assumption is supported by the literature [31,32].

For each source with a given polarity and popularity, we pick the required number of individuals with polarity values in an interval around the source polarity. For instance, if a source has popularity of 24 and polarity of 0.5, we will pick the 24 users whose polarity is closest to 0.5 and we will connect them to the source.

#### 2.3.2. Social network

Users connect to each other in a social network. The social network is the channel through which users are exposed to news items from sources they are not following.

We aim at creating a social network with realistic characteristics. For this reason, we generate it via an Lancichinetti–Fortunato–Radicchi (LFR) benchmark^6^ [33]. The LFR benchmark ensures that the social network has a community structure, a broad degree distribution, and communities are overlapping, i.e. they can share nodes. All these characteristics are typical of real-world social networks. We fix the number of nodes to ≈16 000, while the number of communities is variable and not fixed by the LFR’s parameters.

We need an additional feature in the social network: polarity homophily. People are more likely to be friends with like-minded individuals. This is supported by studies of politics on social media [34]. We ensure homophily by iterating over all communities generated by the LFR benchmark and assigning to users grouped in the same community a portion of the polarity distribution.

For instance, if a community includes 12 nodes, we take 12 consecutive values in the polarity distribution and we assign them to the users. This procedure generates extremely high polarity assortativity. The Pearson correlation of the polarity values at the two endpoints of each edge is ≈0.89.

### 2.4. Actions

A news source publishes to all the users following it an item *i* carrying the source’s polarity *p*_*i*_ and truthfulness *t*_*i*_. Every time a user sees an item *i*, it calculates how acceptable the item is, using the function *f*_*i*,*u*_. An item is acceptable if it is (i) truthful and (ii) it is not far from the user in the polarity spectrum—experiments [35] show how this is a reasonable mechanics: users tend to trust more sources with a similar polarity to their own. Mathematically, (i) means that *f*_*i*,*u*_ is directly proportional to *t*_*i*_; while (ii) means that *f*_*i*,*u*_ is inversely proportional to the difference between *p*_*i*_ and *p*_*u*_

$$f_{i,u}=\frac{t_{i}}{\left|p_{i}-p_{u}\right|}.$$

The acceptability function *f*_*i*,*u*_ has two issues: first, its domain spans from 0 (if *t*_*i*_ = 0) to +∞ (if *p*_*i*_ = *p*_*u*_). This can be solved by the standard transformation *x*/(*x* + 1), which is always between 0 and 1 if *x* ≥ 0.

Second, for the discussion of our parameters and results, it is more convenient to estimate a degree of ‘unacceptability’, which is the opposite of the acceptability *f*_*i*,*u*_. This can be achieved by the standard transformation 1 − *x*. Putting the two transformations together, the unacceptability $\bar{\;f_{i,u}}$ of item *i* for user *u* is

$$\bar{\;f_{i,u}}=1-\frac{\;f_{i,u}}{f_{i,u}+1}.$$

Users have a finite tolerance for how unacceptable a news item can be. If the item exceeds this threshold, meaning $\bar{\;f_{i,u}}>\phi$, the user will flag the item. On the other hand, if the news item has low to zero unacceptability, meaning $\bar{\;f_{i,u}}<\rho$, the user will reshare it to their friends. If $\rho \leq \bar{\;f_{i,u}}\leq \phi$, the user will neither flag nor reshare the item.

The parameters *ϕ* and *ρ* regulate which and how many news items are flagged, and thus we need to tune them to generate realistic results—as we do in the Results section.

### 2.5. Monopolar model

The monopolar model is the result of removing everything related to polarity from the bipolar model. The sharing and flagging criteria are the same as in the bipolar model—testing $\bar{\;f_{i,u}}$ against the *ρ* and *ϕ* parameters, with the difference being in how $\bar{\;f_{i,u}}$ is calculated. The unacceptability of a news item is now simply the opposite of its truthfulness, i.e. $\bar{\;f_{i,u}}=1-t_{i}$.

Moreover, in the monopolar model users connect to random news sources and there is no polarity homophily in the social network.

The monopolar model attempts to reproduce the assumption of real-world crowdsourced flagging systems: only the least truthful articles are flagged. However, we argue that it is not a good representation of reality because truthfulness assessment is not an objective process: it is a subjective judgement and it includes pre-existing polarization of both sources and users. The bipolar model can capture such polarization while the monopolar model cannot.

### 2.6. Example

**Figure 4.**
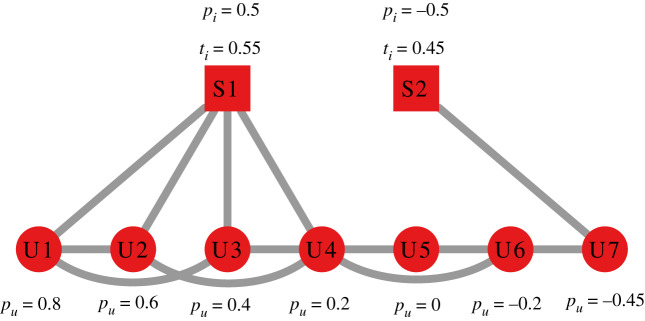
Two simple structures with sources (squares) and users (circles). Edges connect sources to the users following them and users to their friends. Each source has an associated *t*_*i*_ and *p*_*i*_ value and each user has an associated *p*_*u*_ value next to their respective nodes.

To understand what happens in the bipolar and monopolar models, consider figure 4 as a toy example. Table 2*a*,*b* calculates $\bar{\;f_{i,u}}$ for all user–source pairs in the bipolar and monopolar models, respectively. Table 3*a*,*b* counts the number of flags received by each source for different combinations of the *ρ* and *ϕ* parameters in the bipolar and monopolar models, respectively. A few interesting differences between the bipolar and monopolar models appear.

**Table 2.** The $\bar{\;f_{i,u}}$ value for each user–source pair from figure 4 in the (*a*) bipolar and (*b*) monopolar models.

(*a*) bipolar’s $\bar{\;f_{i,u}}$			(*b*) monopolar’s $\bar{\;f_{i,u}}$		
user	S1	S2	user	S1	S2
U1	0.35	0.74	U1	0.45	0.55
U2	0.15	0.71	U2	0.45	0.55
U3	0.15	0.66	U3	0.45	0.55
U4	0.35	0.61	U4	0.45	0.55
U5	0.48	0.52	U5	0.45	0.55
U6	0.56	0.40	U6	0.45	0.55
U7	0.62	0.10	U7	0.45	0.55

**Table 3.** The number of flags each source in figure 4 gets in the (*a*) bipolar and (*b*) monopolar models, for varying values of *ρ* and *ϕ*.

(*a*) bipolar				(*b*) monopolar			
*ρ*	*ϕ*	S1	S2	*ρ*	*ϕ*	S1	S2
0.67	0.7	0	2	0.67	0.7	0	0
0.57	0.6	1	1	0.57	0.6	0	0
0.49	0.54	1	1	0.49	0.54	0	1
0.36	0.44	2	0	0.36	0.44	4	1
0.2	0.3	2	1	0.2	0.3	4	1
0.1	0.6	0	0	0.1	0.6	0	0
0.1	0.5	0	0	0.1	0.5	0	1
0.1	0.14	4	0	0.1	0.14	4	1

In the monopolar model, only the direct audience of a source can flag its news items and, if one member of the direct audience flags, so will all of them. This is because $\bar{\;f_{i,u}}$ is equal for all nodes, thus either $\bar{\;f_{i,u}}>\phi$ and the entire audience will flag the item (and no one will reshare it) or $\bar{\;f_{i,u}}<\rho$ and the entire network—not just the audience—will reshare the item, and no one will ever flag it.

This is not true for the bipolar model. S1 (figure 4) can be either flagged by its entire audience (*ϕ* = 0.14); by part of its audience (*ϕ* = 0.3); or by nodes who are not in its audience at all (users U5 and U6 for *ϕ* = 0.44; or user U7 for *ϕ* = 0.6). On the other hand, in our examples, S2 is never flagged by its audience (U7). When S2 is flagged, it is always because it percolated to a user for which $\bar{\;f_{i,u}}>\phi$, via a chain of users for which $\bar{\;f_{i,u}}<\rho$, because $\bar{\;f_{i,u}}$ is not constant across users any longer.

## 3. Results

### 3.1. Parameter tuning

Before looking at the results of the model, we need to identify the range of parameter values that can support robust and realistic results. The most important of the two parameters is *ϕ*, because it determines the number of flags generated in the system.

**Figure 5.**
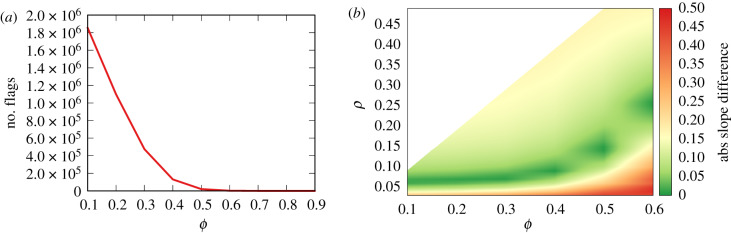
(*a*) The number of flags (*y*-axis) in the bipolar model for different values of *ϕ* (*x*-axis). (*b*) The slope difference (colour; red = high, green = low) between the real world and the bipolar fit between the source popularity and the number of flags received, per combination of *ϕ* and *ρ* values (*x*–*y* axis).

Figure 5*a* shows the total number of flags generated per value of *ϕ*. As expected, the higher the *ϕ*, the fewer the flags, as the user finds more news items acceptable. The sharp drop means that, for *ϕ* > 0.6, we do not have a sufficient number of flags to support our observation of the model’s behaviour. Thus, hereafter, we will only investigate the behaviour of the model for *ϕ* ≤ 0.6.

*ρ* is linked to *ϕ*; specifically, its value is capped by *ϕ*. A world with *ρ* ≥ *ϕ* is unreasonable, because it would be a scenario where a user feels enough indignation by an item that they will flag it, but then they will also reshare it to their social network. Thus, we only test scenarios in which *ρ* < *ϕ*.

Another important question is what combination of *ϕ* and *ρ* values generates flags that can reproduce the observed relation between source popularity and the number of flags we see in figure 1. To do so, we perform a grid search, testing many combinations of *ϕ*–*ρ* values. Our quality criterion is the absolute difference in the slope of the power fit between popularity and the number of flags. The lower the difference, the better the model is able to approximate reality.

Figure 5*b* shows such a relationship. We can see that there is an area of high performance at all levels of *ϕ*.

### 3.2. Bipolar model

**Figure 6.**
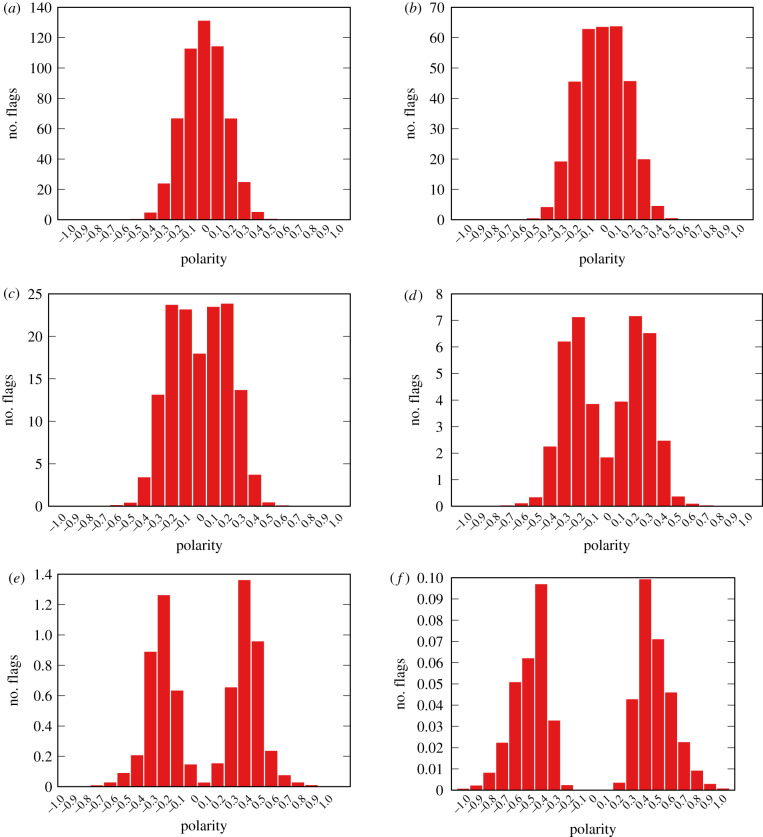
Flag count per polarity of items at different flaggability thresholds *ϕ* for the bipolar model. Reshareability parameter *ρ* = 0.08. Average of 50 runs. (*a*) *ϕ* = 0.1, (*b*) *ϕ* = 0.2, (*c*) *ϕ* = 0.3, (*d*) *ϕ* = 0.4, (*e*) *ϕ* = 0.5 and (*f*) *ϕ* = 0.6.

Figure 6 shows the distribution of the polarity of the flagged news items, for different values of *ϕ* and setting *ρ* = 0.08, an interval including the widest spectrum of goodness of fit as shown in figure 5*b*. We run the model 50 times and take the average of the results, to smooth out random fluctuations.

We can see that our hypothesis is supported: in a polarized environment the vast majority of flagged news items are neutral. This happens for *ϕ* ≤ 0.3, which, as we saw in figure 5*b*, is the most realistic scenario. For *ϕ* ≥ 0.4, our hypothesis would not be supported, but, as we can see in figure 5*b*, this is the area in red, where the model is a bad fit for the observations anyway—since here we are looking at *ρ* = 0.08 results.

**Figure 7.**
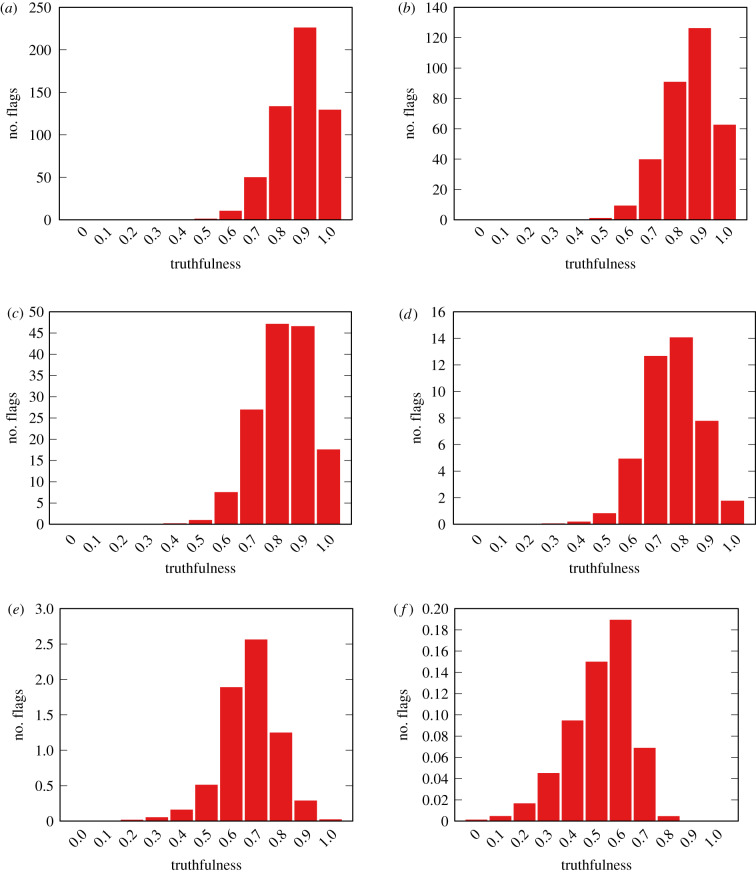
Flag count per truthfulness of items at different flaggability thresholds *ϕ* for the bipolar model. Reshareability parameter *ρ* = 0.08. Average of 50 runs. (*a*) *ϕ* = 0.1, (*b*) *ϕ* = 0.2, (*c*) *ϕ* = 0.3, (*d*) *ϕ* = 0.4, (*e*) *ϕ* = 0.5 and (*f*) *ϕ* = 0.6.

Figure 7 shows the distribution of truthfulness of the flagged items. These distributions show that, by flagging following their individual polarization, users in the bipolar model end up flagging the most truthful item they can—if *ϕ* is high enough, items with *t*_*i*_ ∼ 1 cannot be flagged almost regardless of the polarity difference.

The two observations put together mean that, in the bipolar model, the vast majority of flags come from extremists who are exposed to popular neutral and truthful news. The extremists do not follow the neutral and truthful news sources, but get in contact with neutral and truthful viewpoints because of their social network.

The bipolar model results—in accordance with the observation from figure 1—suggest that more popular items are shared more and thus flagged more. One could be tempted to identify and remove fake news items by taking the ones receiving more than their fair shares of flags given their popularity. However, such a simple system would not work in reality. Figure 1 is based on data coming after Facebook’s machine learning pre-processor, the aim of which is to minimize false positives.^7^ Thus, even after controlling for a number of factors—source popularity, reputation, etc.—most reported flags still end up attached to high-popularity, high-reputability sources.

### 3.3. Monopolar model

**Figure 8.**
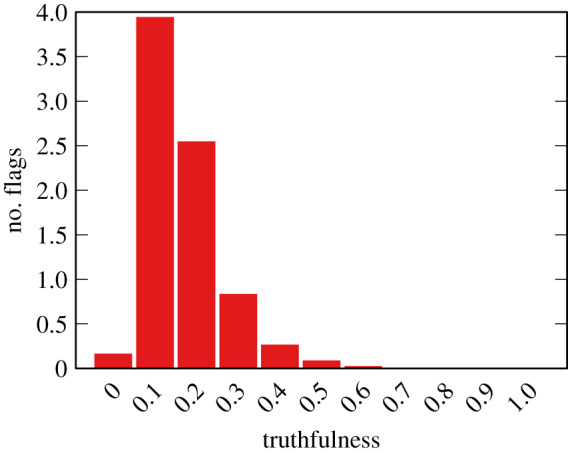
Flag count per truthfulness of items for the monopolar model for *ϕ* = 0.6. Average of 50 runs.

In the monopolar model, we remove all aspects related to polarity, thus we cannot show the polarity distribution of the flags. Moreover, as we have shown in §2.6, the effect of *ρ* and *ϕ* is marginal. Thus we only show in figure 8 the truthfulness distribution of the flags, for only *ϕ* = 0.1 and *ρ* = 0.08, noting that all other parameter combinations result in a practically identical distribution.

The monopolar results show the flag truthfulness distribution as the ideal result. The distribution shows a disproportionate number of flags going to low truthfulness news items, as they should—the drop for the lowest truthfulness value is due to the fact that there are few items at that low level of truthfulness, and that they are not reshared.

Is this ideal result realistic? If we use the same criterion as we used for the bipolar model to evaluate the quality of the monopolar model, the answer is no. The absolute slope difference in the popularity–flag regression between observation and the monopolar model is ≈0.798 for all *ϕ*–*ρ* combinations. This is a significantly worse performance than the worst-performing versions of the bipolar model—figure 5*b* shows that no bipolar version goes beyond a slope difference of 0.5.

Thus we can conclude that the monopolar model is not a realistic representation of reality, even if we would expect it to correctly flag the untruthful news items. The bipolar model is a better approximation, and results in flagging truthful news items.

### 3.4. Robustness

**Figure 9.**
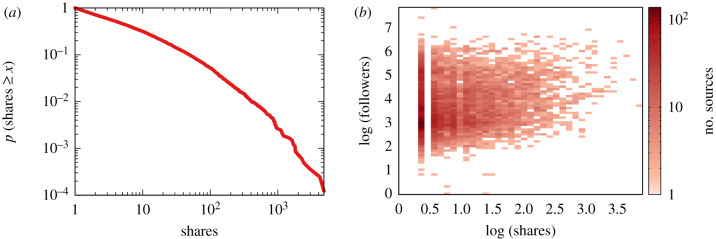
(*a*) The cumulative distribution of source activity in Facebook in our dataset: the probability (*y*-axis) of a news source sharing a given number of items or more (*x*-axis). (*b*) The relationship between activity (*x*-axis) and popularity (*y*-axis) in our Facebook dataset.

Our bipolar model makes a number of simplifying assumptions that we need to test. First, we are showing results for a model in which all news sources have the same degree of activity, meaning that each source will publish exactly one news item. This is not realistic: data from Facebook pages show that there is a huge degree of activity heterogeneity (figure 9*a*).

There is a mild positive correlation between the popularity of a page and its degree of activity (log-log Pearson correlation of ≈0.12; figure 9*b*). For this reason, we use the real-world distribution of page popularity and we lock it in with its real-world activity level. This is the weighted bipolar model, in which each synthetic news source is the model’s equivalent of a real page, with its popularity and activity.

A second simplifying assumption of the bipolar model is that the reshareability and flaggability parameters *ρ* and *ϕ* are the same for every individual in the social network. However, people might have different trigger levels. Thus we create the variable bipolar model, where each user has its own *ρ*_*u*_ and *ϕ*_*u*_. These values are distributed normally, with their average $\bar{\rho }=0.08$ (and standard deviation 0.01) and $\bar{\;\phi }$ depending on which average value of *ϕ* we are interested in studying (with the standard deviation set to one-eighth of $\bar{\;\phi }$).

**Figure 10.**
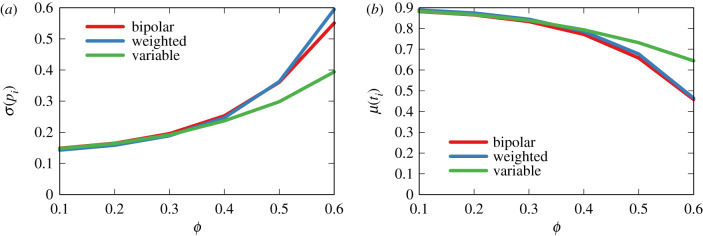
Dispersion of polarization (*a*) and average truthfulness (*b*) of the flagged items in the bipolar model and its weighted and variable variants.

Figure 10 shows the result of the weighted and variable variants against the original bipolar model. In figure 10*a*, we report the dispersion (standard deviation) of the polarization values of the flags. A low dispersion means that flags cluster in the neutral portion of the polarity spectrum, meaning that most flags signal neutral news items. In figure 10*b*, we report the average truthfulness of flagged items.

We can see that taking into account the pages’ activities increases the dispersion by a negligible amount and only for high values of *ϕ*. This happens because there could be some extremely active fringe pages spamming fake content, which increases the likelihood of extreme flags. There is no difference in the average truthfulness of flagged items.

Having variable *ϕ* and *ρ* values, instead, actually decreases dispersion, making the problem worse—although only for larger values of *ϕ*. In this configuration, a very tolerant society with high (average) *ϕ* would end up flagging mostly neutral reporting—as witnessed by the higher average truthfulness of the reported items. This is because lower-than-average *ρ*_*u*_ users will be even less likely to reshare the most extreme news items.

**Figure 11.**
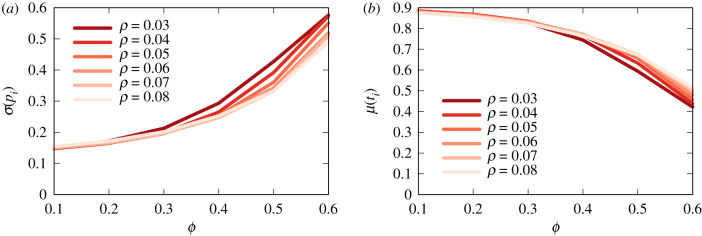
Dispersion of polarization (*a*) and average truthfulness (*b*) of the flagged items for different values of reshareability *ρ*.

So far we have kept the reshareability parameter constant at *ρ* = 0.08. If we change *ρ* (figure 11) the dispersion of a flag’s polarity (figure 11*a*) and its average truthfulness value (figure 11*b*) do not significantly change. The changes are due to the fact that *ρ* simply affects the number of flags: a higher *ρ* means that users are more likely to share news items. More shares imply more news items percolating through the social network and thus more flags.

The bipolar model contains many elements besides the *ρ* and *ϕ* parameters. For instance, it imposes that the social network has several communities and that social relationships are driven by homophily. These two elements are based on existing literature, yet we should test their impact on the model.

First, keeping everything else constant, the no-homophily variant allows users to connect to friends ignoring their polarity value. In other words, polarity is randomly distributed in the network. Second, keeping everything else constant, the no-community variant uses an Erdős–Rényi random graph as the social network instead of an LFR benchmark. The Erdős–Rényi graph extracts connections between nodes uniformly at random and thus it has, by definition, no community structure.

**Figure 12.**
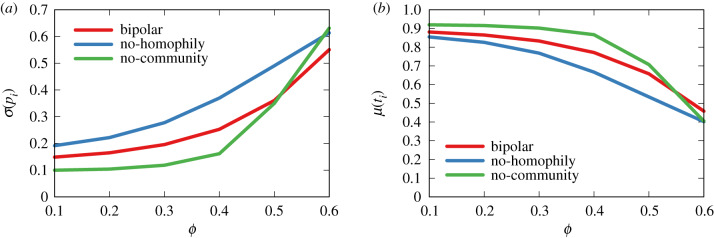
Dispersion of polarization (*a*) and average truthfulness (*b*) of the flagged items in the bipolar and alternative models.

Figure 12 shows the impact on flag polarity dispersion (figure 12*a*) and average truthfulness (figure 12*b*). The no-homophily variant of the bipolar model has a significantly higher dispersion in the flag polarity distribution, and lower truthfulness average, and the difference is stable (though stronger for values of *ρ* above 0.3). This means that polarity homophily is playing a key role in ensuring that flags are predominantly assigned to neutral news items: if we remove it, the accuracy in spotting fake news increases.

In contrast, removing the community structure from the network will result in a slightly smaller dispersion of flag’s polarity and higher average flag truthfulness. The lack of communities might cause truthful items to spread more easily, and thus be flagged, increasing the average flag truthfulness.

## 4. Discussion

In this paper, we show how the assumption of traditional crowdsourced content policing systems is unreasonable. Expecting users to flag content carries the problematic assumption that a user will genuinely attempt to estimate the veracity of a news item to the best of their capacity. Even if that was a reasonable expectation to have, a user’s estimation of veracity will be made within their individual view of the world and variable polarization. This will result in assessments that will give an easier pass to biased content if they share such bias. This hypothesis is supported by our bipolar agent-based model. The model shows that even contexts that are extremely tolerant towards different opinions, represented by our flaggability parameter *ϕ*, would still mostly flag neutral content, and produce results that fit well with observed real-world data. Moreover, by testing the robustness of our model, we show how our results hold both for the amount of heterogeneity of source activity and for individual differences in both tolerance and propagation attitudes.

Removing polarization from the model, and thus testing what we defined as the monopolar model, attempts to reproduce the assumptions that would make a classical content policy system work. The monopolar model, while seemingly based on reasonable assumptions, is not largely supported by established literature in the area of online behaviour and social interaction, differently from the bipolar model. Moreover, it is not able to deliver on its promises in terms of ability to represent real-world data.

Our paper has a number of weaknesses and possible future directions. First, our main results are based on a simulated agent-based model. The results hold as long as the assumptions and the dynamics of the models are an accurate approximation of reality. We provided evidence to motivate the bipolar model’s assumptions, but there could still be factors unaccounted for, such as the role of originality [36] or of spreaders’ effort [37] in making content go viral. Second, many aspects of the model were fixed and should be investigated. For instance, there is a strong polarity homophily between users and news sources, and in user–user connections in the social network. We should investigate whether such strong homophily is really supported in real-world scenarios. Third, the model has an essentially static structure. The users will never start/stop following news sources, nor befriend/unfriend fellow users. Such actions are common in real-world social systems and should be taken into account. Fourth the model only assumes news stories worth interacting with. This is clearly different from the reality where, in a context of overabundant information, most stories are barely read and collect few reshares or flags. Including those news stories in the model could certainly affect the overall visibility of other items. Finally, the model does not take into account reward and cost functions for both users and news sources. What are the repercussions for a news source of having its content flagged? Should news sources attempt to become mainstream and gather following? Such reward/cost mechanisms are likely to greatly influence our outcomes. We plan to address the last two points in future expansions of our model.

## Endnotes

1 https://www.facebook.com/facebookmedia/blog/working-to-stop-misinformation-and-false-news (April 2017, date of access 3 March 2020).

2 https://socialscience.one/blog/unprecedented-facebook-urls-dataset-now-available-research-through-social-science-one (February 2020, date of access 3 March 2020).

3 From a least-squares fit in a log-log space. Alternative hypotheses such as linear relationship or exponential relationship are discarded, with *p*-values approximately 0.98 and 0.34, respectively.

4 https://www.crowdtangle.com/

5 https://electionstudies.org/resources/anes-guide/top-tables/?id=29 (date of access 11 November 2019).

6 https://sites.google.com/site/andrealancichinetti/files

7 https://about.fb.com/news/2018/06/increasing-our-efforts-to-fight-false-news/ (date of access 7 January 2020).

## Data accessibility

The archive containing the data and code necessary for the replication of our results can be found at http://www.michelecoscia.com/wp-content/uploads/2020/03/20200304_ffff.zip
